# Expanding the genetic spectrum of choroideremia in an Australian cohort: report of five novel *CHM* variants

**DOI:** 10.1038/s41439-020-00122-w

**Published:** 2020-10-23

**Authors:** Terri L. McLaren, John N. De Roach, Jennifer A. Thompson, Fred K. Chen, David A. Mackey, Ling Hoffmann, Isabella R. Urwin, Tina M. Lamey

**Affiliations:** 1 Australian Inherited Retinal Disease Registry and DNA Bank, Department of Medical Technology and Physics, Sir Charles Gairdner Hospital, Hospital Avenue, Nedlands, Western Australia Australia; 2 Centre for Ophthalmology and Visual Science, The University of Western Australia, 35 Stirling Highway, Crawley, Perth, Western Australia Australia; 3 Lions Eye Institute, 2 Verdun Street, Nedlands, Western Australia Australia; 4 Department of Ophthalmology, Royal Perth Hospital, Victoria Square, Perth, Western Australia Australia; 5 Department of Ophthalmology, Perth Children’s Hospital, Hospital Avenue, Nedlands, Western Australia Australia

**Keywords:** Medical genetics, Genotype

## Abstract

Choroideremia is an X-linked chorioretinal dystrophy caused by mutations in the *CHM* gene. Several *CHM* gene replacement clinical trials are in advanced stages. In this study, we report the molecular confirmation of choroideremia in 14 Australian families sourced from the Australian Inherited Retinal Disease Registry and DNA Bank. Sixteen males (14 symptomatic) and 18 females (4 symptomatic; 14 obligate carriers) were identified for analysis. Participants’ DNA was analyzed for disease-causing *CHM* variants by Sanger sequencing, TaqMan qPCR and targeted NGS. We report phenotypic and genotypic data for the 14 symptomatic males and four females manifesting disease symptoms. A pathogenic or likely pathogenic *CHM* variant was detected in all families. Eight variants were previously reported, and five were novel. Two *de novo* variants were identified. We previously reported the molecular confirmation of choroideremia in 11 Australian families. This study expands the *CHM* genetically confirmed Australian cohort to 32 males and four affected carrier females.

## Eye disease: Finding new gene variants for choroideremia

Additional variants of the gene responsible for choroideremia, a rare genetic disease that affects blood flow in the eye, have been identified. Choroideremia is X-linked, which means it is much more likely to manifest in males, who do not have a second X chromosome that could carry a functional gene copy. Over time, choroideremia can cause blindness. Terri McLaren at Sir Charles Gairdner Hospital in Perth, Australia, and co-workers aimed to identify new genetic variants that cause choroideremia. Using gene sequencing technology, they identified affected individuals in 14 Australian families, including five new and eight known variants. Prior to sequencing, other eye diseases (excluding choroideremia) had been diagnosed in over one-third of the families, indicating that choroideremia is underdiagnosed. These results will help to build a database of patients for future genetic and other therapies.

## Introduction

Choroideremia (CHM, OMIM: 303100) is a chorioretinal dystrophy inherited in an X-linked recessive manner with an incidence between 1:50,000^1^ and 1:100,000^2^. It is characterized by progressive degeneration of the retinal pigment epithelium (RPE), photoreceptors, and choroid^3^. Individuals with choroideremia usually present with a distinctive fundus appearance, featuring a scalloped choroid due to atrophy of the choroidal vessels^4^.

Choroideremia is caused by mutations in the *CHM* gene (OMIM: 300390), which is located at Xq21.2 and comprises 15 exons^5^ encoding Rab escort protein 1 (REP-1). Currently, 293 disease-causing variants in the *CHM* gene are listed in the Human Genome Mutation Database^6^.

Due to the monogenic nature and distinctive phenotype of this disease, direct sequencing of the *CHM* gene, with follow-up deletion/duplication analysis where required, has been highly effective for genetic confirmation of clinically diagnosed individuals^7^. However, next-generation sequencing (NGS) has sometimes unexpectedly identified *CHM* mutations in male and female individuals with an alternative clinical diagnosis, such as retinitis pigmentosa (RP)^8–10^. Thus, choroideremia may have a variable phenotype, leading to underreporting^11^.

Clinical trials for therapeutic gene replacement of the *CHM* gene are at an advanced stage. Following the first of these trials (NCT01461213)^12^, further phase 1/2 trials are complete or underway. A phase 3 trial (NCT03496012) is underway for 140 participants across clinical sites in the United States, Canada, Europe, and the United Kingdom^13^. No *CHM* gene therapy trials are underway in Australia.

The Australian Inherited Retinal Disease Registry and DNA Bank (AIRDR) previously genetically confirmed choroideremia in individuals from 11 Australian families^7^. The primary aim of the present study was to genetically characterize recently recruited pedigrees to update the spectrum and prevalence of *CHM* mutations in Australian families and to further consolidate potential candidates for future gene-specific clinical trials or treatments. Here, we identified 16 additional males with genetically confirmed choroideremia, two of whom are currently asymptomatic, and 14 asymptomatic carrier females sourced from 14 families. We also identified four female carriers with a vision-threatening phenotype. Such female patients are also relevant to gene-specific clinical trials or treatments but tend to be overlooked.

In the cohort described in our previous study, we genetically confirmed 16 affected males and 12 asymptomatic carrier females sourced from 11 families. Here, we present the combined mutation spectrum, which includes five novel *CHM* mutations, for all 32 genetically confirmed males from 25 Australian families.

## Methods

### Research participants

Participants were identified from the AIRDR^14^. Interrogation of the registry identified nine pedigrees not previously reported with at least one individual clinically diagnosed with choroideremia. Three additional pedigrees, each containing one participant clinically diagnosed with RP, were included in this study where previous analyses revealed potentially disease-causing *CHM* variants. Two families were added after testing negative for the X-linked RP genes *RP2* and *RPGR*, resulting in a suspected diagnosis of choroideremia. In all, 34 participants from 14 families were included in this present study. They comprised 14 symptomatic males, two asymptomatic males, four affected females of varying clinical severity and 14 unaffected, suspected carrier females. For 10 out of 14 families, the proband was a male with clinical features consistent with choroideremia. For two families, there were no consenting affected male participants in the registry. These families are identified here by the numbers 12–25 to distinguish them from families 1 to 11 in our previously published study^7^.

### Genetic analyses

DNA samples were collected, processed, and stored, as previously described^14,15^.

Proband DNA was analyzed using various methods (Table 1).

**Table 1:** Genetic analysis methods used for 34 study participants.

Family member	Status	Sex	Relationship to proband	Service provider	Analysis method	Comment
12-1	Affected	F	Proband	AGRF	Targeted sequencing of familial *CHM* variant	Targeted sequencing based on externally provided familial genetic data
13-1	Affected	M	Proband	CEI	Targeted NGS of ocular genes (Retinal Dystrophy Panel v8; 244 genes) TaqMan qPCR analysis (exons 4 and 9 sampled)	*CHM* variant first identified via targeted panel NGS (performed given the participant’s diagnosis of retinitis pigmentosa).
13-2	Affected	M	Brother	AGRF MVL	Sanger sequencing of *CHM* TaqMan qPCR analysis	
13-3	Obligate carrier	F	Mother	CEI	TaqMan qPCR analysis	
14-1	Affected	M	Proband	CEI	Sanger sequencing of *CHM*	
14-2	Affected	M	Maternal uncle	CEI	Targeted sequencing of familial *CHM* variant	
14-3	Obligate carrier	F	Mother	CEI	Targeted sequencing of familial *CHM* variant	
14-4	Obligate carrier; Affected	F	Maternal aunt	AGRF	Targeted sequencing of familial *CHM* variant	
14-5	Obligate carrier	F	Maternal aunt	AGRF	Targeted sequencing of familial *CHM* variant	
15-1	Affected	M	Proband	CEI	Targeted NGS of ocular genes (Retinal Dystrophy Panel v6; 226 genes)	*CHM* variant first identified via targeted NGS (performed given the participant’s diagnosis of retinitis pigmentosa)
15-2	Affected	M	Brother	AGRF	Targeted sequencing of familial *CHM* variant	
15-3	Obligate carrier	F	Daughter	MVL	Targeted sequencing of familial *CHM* variant	
16-1	Affected	F	Proband	CEI	Sanger sequencing of *CHM*	Clinically diagnosed as manifesting *CHM* carrier. No affected males identified in family.
17-1	Affected	M	Proband	AGRF MVL	Sanger sequencing of *CHM* Confirmational targeted sequencing	
17-2	Obligate carrier	F	Mother	AGRF MVL	Targeted sequencing of familial *CHM* variant Confirmational targeted Sanger sequencing	Proband variant not detected; assumed *de novo*
18-1	Affected	M	Proband	AGRF	Targeted sequencing for confirmation of variant	Based on externally provided results
18-2	Obligate carrier	F	Mother	AGRF	Targeted sequencing of familial *CHM* variant	
18-3	Obligate carrier	F	Daughter	AGRF	Targeted sequencing of familial *CHM* variant	
19-1	Obligate carrier	F	Proband	AGRF	Targeted sequencing of familial *CHM* variant	Based on externally provided results
19-2	Affected	M	Son	AGRF	Targeted sequencing of familial *CHM* variant	Based on externally provided results; asymptomatic
20-1	Affected	M	Proband	AGRF	Targeted sequencing of familial *CHM* variant	Based on externally provided results
20-2	Affected	M	Brother	AGRF	Targeted sequencing of familial *CHM* variant	
20-3	Obligate carrier	F	Mother	AGRF	Targeted sequencing of familial *CHM* variant	
20-4	Obligate carrier	F	Maternal aunt	AGRF	Targeted sequencing of familial *CHM* variant	Confirmation of externally provided results
21-1	Affected	M	Proband	MVL	Sanger sequencing of *CHM* TaqMan qPCR analysis	
21-2	Obligate carrier	F	Mother	MVL	Targeted sequencing of familial *CHM* variant	
22-1	Affected	M	Proband	AGRF MVL	Sanger sequencing of *CHM* Confirmational Sanger sequencing	
22-2	Obligate carrier	F	Mother	MVL	Targeted sequencing of familial *CHM* variant	Proband variant not detected; assumed *de novo*
23-1	Affected	M	Proband	MVL	Sanger sequencing of *CHM*	Analyzed first for variants in XLRP genes, *RP2* and *RPGR*
23-2	Obligate carrier	F	Mother	MVL	TaqMan qPCR analysis	
24-1	Affected	F	Proband	MVL	Targeted NGS of ocular genes (Vision Panel v1; 537 genes)	*CHM* variant first identified via targeted NGS (performed given the participant’s initial diagnosis of retinitis pigmentosa)
24-2	Affected	M	Son	MVL	Targeted sequencing of familial *CHM* variant	Asymptomatic
25-1	Affected	M	Proband	MVL	Sanger sequencing of *CHM*	Analyzed first for variants in XLRP genes, *RP2* and *RPGR*
25-2	Obligate carrier	M	Mother	MVL	Targeted sequencing of the familial *CHM* variant	

AGRF Australian Genome Research Facility.

CEI Casey Eye Institute.

MVL Molecular Vision Laboratory.

XLRP X-linked retinitis pigmentosa.

Gene Reference sequence utilized NM_000390.2; NM_000390.3 (GRCh37).

Proband DNA was analyzed by Sanger sequencing of all 15 exons and flanking intronic regions of the *CHM* gene (Molecular Vision Laboratory (MVL), Oregon, or Australian Genome Research Facility (AGRF), Perth). Where a candidate disease variant was not detected, the possibility of a large deletion/duplication was investigated by TaqMan quantitative PCR (qPCR) (MVL or Casey Eye Institute (CEI), Oregon). Targeted Sanger sequencing was used to verify detected variants, where required, and for testing familial variants in family members.

RefSeq Accession NM_000390.2/3 was used in genetic analyses. Nucleotide 1 corresponds to the A of the ATG translation initiation codon. Sequence variant nomenclature is reported in accordance with the recommendations of the Human Genome Variation Society^16^.

### Classification of variant pathogenicity

The pathogenicity of detected *CHM* variants was ascertained by interrogation of the scientific literature and disease- and locus-specific databases and by *in silico* analysis, as detailed previously^15^. Variant pathogenicity was classified in accordance with recommendations of the American College of Medical Genetics and Genomics and the Association for Molecular Pathology (ACMG)^17^. Splice site variants at consensus dinucleotides (± 1/2) were automatically assigned a pathogenic status^18,19^.

## Results

### Phenotypic data

As the AIRDR is a national registry containing information from participants throughout Australia, phenotypic data such as imaging or electrophysiology results contained in the registry are often incomplete or self-reported. Nevertheless, we report here those phenotypic data that were available.

#### Male participants

At the time of this study, the age range of the 14 affected males was 8–60 years. Their reported ages of onset ranged from 3 to 28 years. All symptomatic males reported night blindness as a presenting feature, with six reporting constricted fields at this time. One male also reported photophobia as a presenting symptom (Table 2).

**Table 2:** Phenotypic information for male and symptomatic female participants in this study.

Family ID	Year recruited	Gender	Current age	Age of onset	Years affected	Age at diagnosis	Onset symptoms			Disease progression	Other comments
							NB	Other	Age	Clinical memorandum	
12-1	2010	F	90	55	35	ND	Yes	ND	**85**:	retinae resemble lacework (CN); vision problems increasing; sees flashes of light; blind in one eye; decreased PV in the other	Strong family history
13-1	2010	M	33	16	17	17	Yes	ND	**17**:	legally blind	Initial diagnosis: RP; myopic; has never driven
									**24**:	no progression of symptoms	
									**32:**	CV OK; PV getting noticeably worse	
13-2	2011	M	35	16	19	20	Yes	ND	**20:**	stopped driving at night	Initial diagnosis: arRP
									**30:**	stopped driving completely	
									**34:**	CV OK; decreased PV; photophobia worse; not legally blind	
14-1	2014	M	12	4	8	7	Yes	ND	**6**:	pigmentation of fundus (CN)	
									**8**:	patchy atrophy, mild attenuation of retinal arteries, slightly swollen optic nerve heads; no visual symptoms except depth perception issues; microperimetry showed slight reduction in retinal sensitivity in central 6^°^(CN)	
14-2	2015	M	23	13	10	20	Yes	ND	**20**:	slow progression; NB worsening in past year	
14-4	2015	F	59	51	8	55	Yes	reduced PV	**51:**	reduced PV	
									**56**:	NB; see flashes of light; faster progression in past few years; stopped driving	
									**59:**	CV and photophobia very bad; no PV	
15-1	2012	M	60	23	37	23	Yes	ND	**30**:	significant deterioration of vision	Initial diagnosis: RP
									**44:**	stopped driving	
									**49**:	LE LP; RE tunnel vision; legally blind	
									**59:**	struggles to see dinner on his plate	
15-2	2012	M	53	28	25	34	Yes	reduced PV	**35**:	legally blind	Initial diagnosis: RP
									**47**:	rapid deterioration in PV	
									**49:**	started using a cane	
16-1	2015	F	72	58	14	ND	Yes	photophobia	**64**:	slow progression; PV not too bad; stopped driving in early morning (glare) and at night; depth perception problems; dry eyes	Isolated case
17-1	2015	M	22	16	6	18	Yes	ND	≤**12**:	no vision problems; slow progression thereafter	Isolated case
									**18**:	VA 6/6 BE; large areas of atrophy in peripheral and perimacular region and defects in RPE suggestive of choroideremia; ERG consistent with choroideremia (CN); NB in last 2-3 years	
18-1	2016	M	44	10	34	41	Yes	photophobia	**28**:	VA 6/6 BE; 15^o^ fields; marked peripheral retinal degeneration, just macula spared in both eyes (CN)	Isolated case; astigmatism; ERG: residual cone function, but extinguished rod response
									**32:**	constriction of visual fields; VA 6/6 BE (CN)	
									**34:**	VA RE 6/7.5 + ; LE 6/6- (CN).	
									**41:**	VA RE 6/12; LE 6/6 (CN); no longer driving; still has functional vision; not deemed legally blind	
19-1	2015	M	14	N/A	N/A	N/A	N/A	N/A	N/A		Asymptomatic male
20-1	2017	M	27	3	24	6	Yes	ND	**16:**	photophobic	Color blind
									**26**:	very slow progression; CV fine; gradual loss in PV; gradual increase in NB; drives in daylight; sees flashes of light & floating spots; light to dark adaptation problems	
20-2	2017	M	25	6	19	17	Yes	reduced PV	**25**:	very slow progression; CV a little blurred; gradual loss of PV; still day/night driving; sees flashes of light & floating spots; light to dark adaptation problems	Myopic
21-1	2016	M	8	4	4	4	Yes	ND	**5**:	no progression as yet; VA 6/9 BE; PV OK; peripheral pigmentary changes; peripheral retinal mottling consistent with choroideremia (CN)	Normal color vision
22-1	2014	M	29	8	21	13	Yes	reduced PV	**21**:	CV OK; stopped driving; LE color perception problems	Isolated case
									**25:**	decreased CV and PV. NB worsened in past few years; LE worse than RE; can still read easily	
									**29:**	photophobia a recent development	
23-1	2010	M	53	21	32	21	Yes	reduced PV	**52:**	very gradual progression; drives in daylight	Initial diagnosis: RP
24-1	2016	F	61	28	33	58	Yes	reduced PV; photophobia	**59:**	VA 3/60 (RE); 6/18 (LE); NB; PV < 3°; photophobic; contrast sensitivity and color test grossly abnormal; flat or grossly reduced ERGs; slow progression (CN)	Initial diagnosis: RCD
24-2	2016	M	26	N/A	N/A	N/A	N/A	N/A	N/A		Asymptomatic male
25-1	2009	M	17	5	12	6	Yes	reduced PV	**10:**	VA RE 6/24; LE 6/30; (CN); no full field ERG responses; PV < 10° (CN)	Initial diagnosis: XLRP

Choroideremia (CHM, OMIM: 303100).

Self-reported information unless indicated otherwise: CN, clinical notes; BE, both eyes; CV, central vision; ERG, electroretinogram; LE, left eye; N/A, not applicable; NB, night blindness; ND, no data; LP, light perception; PV peripheral vision; RCD, rod-cone dystrophy; RE, right eye; RP, retinitis pigmentosa; RPE, retinal pigment epithelium; VA, visual acuity; xl, X-linked.

Initial diagnosis refers to the clinical diagnosis (if not choroideremia) at the start of this study. All ages are presented in years.

Self-reported presenting symptoms were similar among the four families containing more than one symptomatic male (Families 13, 14, 15, and 20), as was age of onset within three families. One exception was Family 14, where self-reported ages of onset differed by nine years (Table 2). This disparity may relate to the generational gap between the proband and his maternal uncle, with the uncle’s existing diagnosis possibly alerting the family to the possibility of disease in the proband.

For each of the two families, family testing revealed the presence of the familial *CHM* mutation in an asymptomatic male with retinal features consistent with choroideremia.

#### Female participants

Fourteen of 18 females in this study were asymptomatic. Four females had reported symptoms of varying severity (Table 2). The ages of onset were in the third (*n* = 1) or sixth (*n* = 3) decade, with night blindness reported as the presenting symptom in all cases, with or without photophobia and/or visual field constriction. Since onset, worsening symptoms have significantly affected their quality of life.

### Genetic results

A pathogenic or likely pathogenic *CHM* variant was identified in all 14 families analyzed (Table 3). All males (14 affected and two asymptomatic) and 16 out of 18 females were hemizygous and heterozygous, respectively, for the detected familial *CHM* variant.

**Table 3:** Disease-causing *CHM* sequence variants identified in the Australian cohort in the present study. Segregation was complete for all families, apart from Families 17 and 22, in which the variants established in the probands are presumed *de novo*.

Family ID	Nucleotide change	Exon/Intron(i)	Predicted protein	Predicted effect^	Novel or Reported	Variant classification (ACMG)
12	c.1358_1359delinsG	11	p.(Ser453*)	Premature truncation of mRNA	Reported	Pathogenic
13	c.(?_-1)_(*1_?)del	1-15	NIL	Entire gene deletion^#^	Reported	Pathogenic
14	c.1584_1587del	13	p.(Val529Hisfs*7)	Premature truncation of mRNA	Reported	Pathogenic
15	c.799 C > T	6	p.(Arg267*)	Premature truncation of mRNA	Reported	Pathogenic
16	c.820-1 G > A	i6	p.?	Abnormal splicing	Novel	Likely pathogenic
17	**c.589dup**	5	p.(Ser197Lysfs*2)	Premature truncation of mRNA	Novel	Pathogenic
18	c.49 + 1 G > T	i1	p.?	Abnormal splicing	Reported	Pathogenic
19	c.1010_1015delinsCA	8	p.(Val337Alafs*6)	Premature truncation of mRNA	Novel	Pathogenic
20	c.1286_1287del	10	p.(Ser429*)	Premature truncation of mRNA	Reported	Pathogenic
21	c.(1770 + 1_1771-1)_(*1962_?)del	15	p.?	Exon 15 deletion^#^	Reported	Pathogenic
22	**c.715** **C** > **T**	6	p.(Arg239*)	Premature truncation of mRNA	Reported	Pathogenic
23	c.(1770 + 1_1771-1)_(*1962_?)del	15	p.?	Exon 15 deletion^#^	Reported	Pathogenic
24	c.767_768del	6	p.(Glu256Valfs*2)	Premature truncation of mRNA	Novel	Pathogenic
25	c.999_1000insT	8	p.(Gln334Serfs*84)	Premature truncation of mRNA	Novel	Pathogenic

**Bolded text** *de novo* variants are denoted in bold.

# deletion breakpoints not identified.

^ where premature truncation of mRNA is predicted, nonsense-mediated decay was considered likely.

The mothers of two affected males did not possess the familial *CHM* variant, suggesting *de novo* events. The apparent *de novo* variants identified in unrelated, isolated males (17-1 and 22-1) are expected to result in premature termination codons (PTCs): the nonsense variant c.715 C > T, p.(Arg239*) has frequently been described in the literature, while the frameshifting duplication c.589dup, p.(Ser197Lysfs*2) is one of 5 novel variants identified in this study.

The frameshift variants c.589dup, p.(Ser197Lysfs*2), c.767_768del, p.(Glu256Valfs*2), c.999_1000insT, p.(Gln334Serfs*84) and c.1010_1015delinsCA, p.(Val337Alafs*6) are predicted to result in protein truncation and nonsense-mediated decay (NMD), with abolition of the protein. Accordingly, these variants have been classified as pathogenic.

The novel splice variant c.820-1 G > A is expected to be pathogenic because it occurs within a canonical splice site. This variant was heterozygous in an affected female proband (16-1) with no known family history of choroideremia. Other pathogenic nucleotide substitutions at this splice acceptor site have been described^20–22^. Notably, a similar *CHM* mutation (c.820-1 G > C) was reported in a female carrier who displayed a highly abnormal RPE without atrophy, with severe loss of visual acuity secondary to a presumed neovascular membrane^20^. In view of the absence of an affected male in Family 16 and this variant not previously described in the literature, we conservatively assessed c.820-1 G > C as likely pathogenic.

Overall, four nonsense, five frameshift and two canonical splice site mutations were detected in 11 families. Gross deletions were identified in the remaining three families, including an entire gene deletion in one family and deletion of exon 15 in two families. Although breakpoints were not identified for the exon 15 deletions, for the purpose of this paper, we have classified them as a single variant. Thus, 13 different causative variants were identified among the 14 families included in this present study.

A genetic diagnosis of choroideremia was therefore confirmed for all nine families with a clinical diagnosis of choroideremia, as well as for the five families with a clinical diagnosis of RP at recruitment, for which no other candidate variants had been detected by previous genetic testing. A clinical re-evaluation of the diagnosis has been made for three of these RP families and is being sought for the other two.

## Discussion

### Present study

In this study, 13 different *CHM* variants classified as pathogenic or likely pathogenic were identified in 14 Australian families. Five variants were novel, and two were *de novo*, including one novel variant.

As in other studies^21,23^, we identified a predominance of causative point mutations. Two gross deletions and one entire gene deletion were detected. Once considered rare, gross deletions now reportedly comprise approximately 20% of disease-causing *CHM* variants^6^. With the identification of an entire gene deletion or deletions of exon 15 among three families, gross deletions now comprise 12% of our combined Australian cohort. Although an exon 15 deletion has been previously reported^24^, the two cases presented in this study are the first reported in an Australian cohort. It is not known whether these families carry the same or distinct nucleotide deletions, as breakpoints were not determined. Similarly, owing to the absence of breakpoint data, it is not known whether the entire *CHM* gene deletion identified in this study is the same as those reported previously.

Entire gene deletions have been reported involving the *CHM* gene alone^21,25,26^ or in various combinations with other genes, which can result in complex syndromic choroideremia phenotypes^11,24,27^. In the absence of breakpoint data for these gross deletions, we cannot establish if they encompass only the *CHM* region or regions and/or regulatory elements of other genes. As associated medical conditions were self-reported as absent in all cases, it is likely that these deletions do not affect the function of other genes.

The clinical features and reported symptoms of carrier females in this study are consistent with the view that females are typically unaffected. Nevertheless, four female participants did show symptoms of varying severity. An underrepresentation of the contribution of *CHM* mutations to disease in affected carrier females may contribute to a diagnosis of RP with autosomal dominant transmission. It is important that severely affected female carriers of X-linked disease be included in considerations regarding inclusion in gene-specific clinical trials or treatments.

Notably, over one-third of the choroideremia-affected pedigrees in the present study were not initially clinically diagnosed with choroideremia. This finding supports the view that choroideremia is underdiagnosed and sometimes misdiagnosed as RP owing to the overlapping clinical features and presenting symptoms of these related conditions and, in some cases, also owing to atypical fundus features or severe phenotype in a female^29^. This highlights the value of nonhypothesis genetic diagnostic testing for suspected RP-affected individuals^11,29,30^.

### Combined studies

The age distribution of the participants in the combined studies, classified by gender and affectation status, is shown in Table 4.

**Table 4:** Demographic information for *CHM* mutation carriers in the combined studies.

	Symptomatic males	Asymptomatic males	Symptomatic females	Asymptomatic females
Number	30	2	4	26
Average current age	37	20	71	55
Age range	8–82	14–26	60–91	12–91
0–10	1	0	0	0
11–20	4	1	0	1
21–30	9	1	0	3
31–40	3	0	0	1
41–50	6	0	0	4
51–60	3	0	0	8
61–70	1	0	2	3
71–80	1	0	1	4
81–90	1	0	0	1
91–100		0	1	1
Deceased	1	0	0	0

Self-reported phenotypic data for individuals with disease-causing *CHM* variants in the combined studies for males and symptomatic females are shown in Supplementary Table 1. One affected male (7-2) was added to our previous study.

The sequence variants established in this study combined with our previous study are detailed in Supplementary Table 2.

In this combined study, we identified a predominance of causative point mutations, including frameshift, nonsense and canonical splice site mutations, with an absence of missense mutations, which is well documented for this gene^21–23,28,31^. Gross deletions were also reported in this study. The preponderance of such mutations suggests that most of the familial variants identified within this updated Australian cohort are likely to be null mutations, as found in other studies^22,28,31^.

Only one mutation, c.1584_1587del (p. Val529Hisfs*7), was detected in both studies. This frameshift variant is thought to occur at a mutation hotspot frequently reported in apparently unrelated pedigrees^9,22,26,28,32–34^.

Of interest, all six nonsense mutations detected across the combined choroideremia cohort of 25 pedigrees are C > T transitions (24% of pedigrees). Five of these are recurrent disease-causing *CHM* variants located at CpG dinucleotides, known mutational hotspots^35,36^. Two were detected in the present study (*de novo* c.715 C > T; c.799 C > T), and three were detected in our previous study (c.757 C > T; c.808 C > T; c.877 C > T). These results reflect the hypermutability for C > T transitions at CpG dinucleotides that occur at these five arginine residues (CGA), resulting in their conversion to a stop codon (TGA), as reported by others^11,22,28,37,38^.

These results indicate that Sanger sequencing of the *CHM* gene in probands with a clinical diagnosis of choroideremia remains an efficient tool in the molecular diagnostic pipeline. In the three families in which a mutation was not detected by Sanger sequencing, follow-up qPCR analysis identified gross deletions in *CHM*. In addition to these reported Australian families, other undiagnosed, untested or unborn male family members may prove to be candidates for future gene therapy.

This Australian cohort now consists of 25 genetically confirmed choroideremia-affected families, with a total of 23 different *CHM* mutations identified.

## Supplementary information

Phenotypic information for both studies

Disease-causing variants for both studies
