# Supplementary material for: Expanding the genetic spectrum of choroideremia in an Australian cohort: report of five novel CHM variants
Source: Hum Genome Var. 2020 Oct 23;7:35. doi: 10.1038/s41439-020-00122-w (PMC7584600; doi:10.1038/s41439-020-00122-w)
Supplement: Supplementary file 1 — Phenotypic information for both studies [file 41439_2020_122_MOESM1_ESM.docx]

Supplementary Table 1: Phenotypic information for symptomatic participants in the combined studies

| Family ID | Year recruited | Gender | Current age | Age of onset | Years affected | Age at diagnosis | Onset symptoms | | Disease Progression | | Other comments |
| --- | --- | --- | --- | --- | --- | --- | --- | --- | --- | --- | --- |
|  |  |  |  |  |  |  | NB | Other | Age | Self-reported/clinical data |  |
| 1-1 | 2009 | M | 29 | Birth | 29 | 7 | Yes | Reduced PV  Photophobia | **7:** fields < 5°  **17:** VA 6/6 BE  **24: v**ery little progression | |  |
| 1-2 | 2009 | M | 27 | Birth | 27 | 5 | Yes | Photophobia | **5:** severely reduced PV | |  |
| 2-1 | 2010 | M | 49 | Birth | 49 | 6 | Yes | Reduced PV | **16: f**ields < 10°  **40:** can still read a bit | |  |
| 3-1 | 2009 | M | 61 | 3 | 58 | 22 | Yes | Reduced PV  Photophobia |  | |  |
| 3-2 | 2010 | M | 17 | 3 | 14 | 4 | Yes | Clumsy |  | |  |
| 4-1 | 2010 | M | 76 | 25 | 51 | 28 | Yes | Blind spot |  | |  |
| 4-2 | 2010 | M | 82 | 35 | 47 | 37 | Yes | Photophobia  Clumsy | **73:** light perception only; central deteriorating faster than peripheral | |  |
| 5-1 | 2011 | M | 49 | 25 | 23 | 27 | Yes | Reduced PV | **28:** legally blind  **41:** PV 8°; severe NB | |  |
| 6-1 | 2012 | M | 44 | 6 | 38 | 3 | Yes | Photophobia | **44:** PV noticeably decreased; still driving in daytime | |  |
| 6-2 | 2014 | M | 46 | 6 | 40 | 3 | Yes | Photophobia | **46:** still driving in daytime | |  |
| 7-1 | 2010 | M | 28 | 5 | 23 | 11 | Yes | Reduced PV | **11:** fields 30° BE  **16:** legally blind; loss of color vision  **19:** fields 10° RE, 15° LE  **22:** fields 5° RE, 11° LE | |  |
| 7-2 | 2019 | M | 20 | 12 | 8 | 15 | Yes |  | **16:** slow deterioration | | Additional family member added to first study |
| 8-1 | 2011 | M | 32 | 8 | 24 | 10 |  | Blind spot | **16:** noticed loss of PV  **25:** PV 8° BE; steady decline in vision | | Initial diagnosis: xlRP |
| 8-2 | 2009 | M | Deceased | ND | ND | 30 |  | Photophobia | **30:** ‘blind’ | | Deceased at 86 yrs |
| 10-1 | 2014 | M | 20 | 1 | 19 | 12 | Yes | Clumsy | **16:** mild deterioration | |  |
| 11-1 | 2014 | M | 42 | 16 | 26 | 17 | Yes |  | **36:** recent steady deterioration; CV blurred; increased photophobia | |  |
| 12-1 | 2010 | F | 90 | 55 | 35 | ND | Yes | ND | **85**: retinae resemble lacework (CN); vision problems increasing; sees flashes of light; blind in one eye; decreased PV in the other | | Strong family history |
| 13-1 | 2010 | M | 33 | 16 | 17 | 17 | Yes | ND | **17**: legally blind  **24**: no progression of symptoms  **32:** CV OK; PV getting noticeably worse | | Initial diagnosis: RP; myopic; has never driven |
| 13-2 | 2011 | M | 35 | 16 | 19 | 20 | Yes | ND | **20:** stopped driving at night  **30:** stopped driving completely  **34:** CV OK; decreased PV; photophobia worse; not legally blind | | Initial diagnosis: arRP |
| 14-1 | 2014 | M | 12 | 4 | 8 | 7 | Yes | ND | **6**: pigmentation of fundus (CN)  **8**: patchy atrophy, mild attenuation of retinal arteries, slightly swollen optic nerve heads; no visual symptoms except depth perception issues; microperimetry showed slight reduction in retinal sensitivity in central 6^º^(CN) | |  |
| 14-3 | 2015 | M | 23 | 13 | 10 | 20 | Yes | ND | **20**: slow progression; NB worsening in past year | |  |
| 14-4 | 2015 | F | 59 | 51 | 8 | 55 | Yes | Reduced PV | **51:** reduced PV  **56**: NB; see flashes of light; faster progression in past few years; stopped driving  **59:** CV and photophobia very bad; no PV | |  |
| 15-1 | 2012 | M | 60 | 23 | 37 | 23 | Yes | ND | **30**: significant deterioration of vision  **44:** stopped driving  **49**: LE LP; RE tunnel vision; legally blind  **59:** struggles to see dinner on his plate | | Initial diagnosis: RP |
| 15-2 | 2012 | M | 53 | 28 | 25 | 34 | Yes | Reduced PV | **35**: legally blind  **47**: rapid deterioration in PV  **49:** started using a cane | | Initial diagnosis: RP |
| 16-1 | 2015 | F | 72 | 58 | 14 | ND | Yes | Photophobia | **64**: slow progression; PV not too bad; stopped driving in early morning (glare) and at night; depth perception problems; dry eyes | | Isolated case |
| 17-1 | 2015 | M | 22 | 16 | 6 | 18 | Yes | ND | **≤12**: no vision problems; slow progression thereafter  **18**: VA 6/6 BE; large areas of atrophy in peripheral and perimacular region and defects in RPE suggestive of choroideremia; ERG consistent with choroideremia (CN); NB in last 2-3 years | | Isolated case |
| 18-1 | 2016 | M | 44 | 10 | 34 | 41 | Yes | Photophobia | **28**: VA 6/6 BE; 15^o^ fields; marked peripheral retinal degeneration, just macula spared in both eyes (CN)  **32:** constriction of visual fields; VA 6/6 BE (CN)  **34:** VA RE 6/7.5+; LE 6/6- (CN).  **41:** VA RE 6/12; LE 6/6 (CN); no longer driving; still has functional vision; not deemed legally blind | | Isolated case; astigmatism;  played squash into teens; ERG: residual cone function, but extinguished rod response |
| 20-1 | 2017 | M | 27 | 3 | 24 | 6 | Yes | ND | **16:** photophobic  **26**: very slow progression; CV fine; gradual loss in PV; gradual increase in NB; drives in daylight; sees flashes of light & floating spots; light to dark adaptation problems | | Color blind |
| 20-2 | 2017 | M | 25 | 6 | 19 | 17 | Yes | Reduced PV | **25**: very slow progression; CV a little blurred; gradual loss of PV; still day/night driving; sees flashes of light & floating spots; light to dark adaptation problems | | Myopic |
| 21-1 | 2016 | M | 8 | 4 | 4 | 4 | Yes | ND | **5**: no progression as yet; VA 6/9 BE; PV OK; peripheral pigmentary changes; peripheral retinal mottling consistent with choroideremia (CN) | | Normal color vision; |
| 22-1 | 2014 | M | 29 | 8 | 21 | 13 | Yes | Reduced PV | **21**: CV OK; stopped driving; LE color perception problems  **25:** decreased CV and PV. NB worsened in past few years; LE worse than RE; can still read easily  **29:** photophobia a recent development | | Isolated case |
| 23-1 | 2010 | M | 53 | 21 | 32 | 21 | Yes | Reduced PV | **52:** very gradual progression; drives in daylight | | Initial diagnosis: RP |
| 24-1 | 2016 | F | 61 | 28 | 33 | 58 | Yes | Reduced PV  Photophobia | **59:** VA 3/60RE; 6/18 LE; NB; PV < 3º; photophobic; contrast sensitivity and color test grossly abnormal; flat or grossly reduced ERGs; slow progression (CN) | | Initial diagnosis: RCD |
| 25-1 | 2009 | M | 17 | 5 | 12 | 6 | Yes | Reduced PV | **10:** VA RE 6/24; LE 6/30; (CN); no full field ERG responses; PV <10º (CN) | | Initial diagnosis: xlRP |

Self-reported information unless indicated otherwise: CN, clinical notes

BE, both eyes; CV, central vision; ERG, electroretinogram; LE, left eye; N/A, not applicable; NB, night blindness; ND, no data; LP, light perception; PV peripheral vision; RCD, rod-cone dystrophy; RE, right eye; RP, retinitis pigmentosa; RPE, retinal pigment epithelium; VA, visual acuity; xl, X-linked.

Initial diagnosis refers to the clinical diagnosis (if not choroideremia) at the start of this study. All ages are presented in years.
