# Supplementary material for: Expanding the genetic spectrum of choroideremia in an Australian cohort: report of five novel CHM variants
Source: Hum Genome Var. 2020 Oct 23;7:35. doi: 10.1038/s41439-020-00122-w (PMC7584600; doi:10.1038/s41439-020-00122-w)
Supplement: Supplementary file 2 — Disease-causing variants for both studies [file 41439_2020_122_MOESM2_ESM.docx]

Supplementary Table 2: Disease-causing *CHM* sequence variants established to date in the Australian Inherited Retinal Disease Registry

| Nucleotide change | Exon/  Intron | Predicted protein | HGMD | Variant classification ACMG | Family ID | Alternative coding nomenclature |
| --- | --- | --- | --- | --- | --- | --- |
| Nonsense |  |  |  |  |  |  |
| c.715C>T | 6 | p.(Arg239*) | CM010795 | Pathogenic | 22 | c.745C>T |
| c.757C>T | 6 | p.(Arg253*) | CM994349 | Pathogenic | 3 | c.787C>T |
| c.799C>T | 6 | p.(Arg267*) | CM970301 | Pathogenic | 15 | c.829C>T |
| c.808C>T | 6 | p.(Arg270*) | CM983732 | Pathogenic | 4 | c.838C>T |
| c.877C>T | 7 | p.(Arg293*) | CM940281 | Pathogenic | 5 | c.907C>T |
| c.1273C>T | 10 | p.(Gln425*) | CM155041 | Pathogenic | 9 | c.1303C>T |
| Frameshift |  |  |  |  |  |  |
| c.589dup | 5 | p.(Ser197Lysfs*2) | - | Pathogenic | 17 | c.589_590insA |
| c.685dup | 5 | p.(Ile229Asnfs*2) | CI155045 | Pathogenic | 7 | c.685_686insA |
| c.767_768del | 6 | p.(Glu256Valfs*2) | - | Pathogenic | 24 | N/A |
| c.999_1000insT | 8 | p.(Gln334Serfs*84) | - | Pathogenic | 25 | N/A |
| c.1010_1015delinsCA | 8 | p.(Val337Alafs*6) | - | Pathogenic | 19 | c.1010_1015delTCATGCinsCA |
| c.1179_1180del | 9 | p.(Phe394Trpfs*23) | CD137318 | Pathogenic | 6 | c.1178_1179delTG |
| c.1286_1287del | 10 | p.(Ser429*) | CD973057 | Pathogenic | 20 | c.1283_1284delTC  c.1313delTC |
| c.1287_1288del | 10 | p.(Glu430Alafs*11) | CD155044 | Pathogenic | 10 | c.1317_1318del |
| c.1358_1359delinsG | 11 | p.(Ser453*) | CX972737 | Pathogenic | 12 | c.1358_1359delCCinsG |
| c.1584_1587del | 13 | p.(Val529Hisfs*7) | CD930932 | Pathogenic | 14; 1 | c.1584_1587delTGTT |
| Gross deletion |  |  |  |  |  |  |
| c.(1770+1_1771-1)_(*1962_?)del ^#^ | 15 | p.? | CG962820^#^ | Pathogenic | 21; 23 | N/A |
| c.(?_-1)_(*1_?)del ^#^ | entire gene | p.? | CG137332^#^ CG1110029^#^ | Pathogenic | 13 | N/A |
| Splicing |  |  |  |  |  |  |
| c.49+1G>T | i1 | p.? | - | Pathogenic | 18 | N/A |
| c.116+1G>A | i2 | p.? | CS032061 | Pathogenic | 2 | c.146+1G>A  IVS2G>A |
| c.819+1G>C | i6 | p.? | CS155042 | Pathogenic | 8 | N/A |
| c.820-1G>A | i6 | p.? | - | Likely pathogenic | 16 | c.850-1G>A |
| c.940+1G>A | i7 | p.? | CS155043 | Pathogenic | 11 | c.970+1G>A |

^#^ It is unknown if the gross deletions identified are novel and distinct from the noted HGMD variants (or each other in the cases of exon 15 deletion) as breakpoints are unknown.
